# Viable bacterial counts of the Pangasius catfish (*Pangasianodon hypophthalmus*), their responses to seasonal variations of physicochemical parameters, and bacterial counts of the cultured ponds

**DOI:** 10.5455/javar.2022.i639

**Published:** 2022-12-31

**Authors:** Md. Nurul Haider, Md. Majharul Islam, Md. Abdul Mukit, Md. Naim Uddin

**Affiliations:** Department of Fisheries Technology, Bangladesh Agricultural University, Mymensingh, Bangladesh

**Keywords:** Physicochemical parameters, seasonal variations, viable bacterial counts, Pangasius catfish, RDA

## Abstract

**Objectives:**

The study was undertaken to evaluate the influences of some physicochemical parameters and viable bacterial counts in cultured ponds (water and sediment) on the viable counts of the Pangasius catfish (*Pangasianodon hypophthalmus*) (gill and intestine) on a seasonal scale.

**Materials and Methods:**

Physicochemical parameters, *viz*., ambient temperature, water temperature, water transparency, pH of the pond waters, and viable bacterial counts of pond water, sediment, fish gills, and intestines, were monitored during four different seasons. The responses of viable counts of bacteria to the seasonal changes of physicochemical parameters were also assessed using redundancy analysis (RDA) and a heatmap coupled with the clustering analysis.

**Results:**

Except for fluctuations in air and water temperatures, the other two physicochemical parameters were almost stable throughout the study periods. The gills and water counts were relatively lower than those of the intestine and sediment. Pearson’s correlation analysis established no significant correlations between the physicochemical parameters and viable bacterial counts. However, significant positive correlations were detected between the viable counts of water and sediment and between the gill and intestine. The RDA plot showed that, except in spring, the viable counts of a particular sample type were similar among the four locations. The results of permutation test showed that, individually none of the studied physicochemical parameters was significant; however, the seasons significantly affected the viable counts.

## Introduction

The number and types of bacteria present in the aquaculture ponds greatly regulate the water quality parameters [1] and are believed to determine the pond dynamics and farming systems as well as the growth and soundness of the farmed organism [2,3]. Bacteria present within the pond environment are mostly populated in the water column and the bottom sediment, and thus fish are always vulnerable to the bacteria available in the pond water and sediment. It is evident that these bacteria modify the bacterial flora of the exterior and interior body parts and organs of fish, including their gills. In addition, the water [4] or food taken by the fish will allow these bacteria to come across the mouth, ultimately going through or settling in their gut [5]. Some of these colonized bacteria are opportunistic pathogens related to fish diseases, especially under stress. Information about the bacterial load and their seasonal variation in pond water, sediment, fish gills, and intestines is essential to recognize and correct abnormal conditions in ponds and fish [6].

Pangasius catfish (*Pangasianodon hypophthalmus*) is one of Bangladesh’s most well-regarded aquaculture species. Production and country-wide availability of their seeds, faster growth, convenient culture technique, higher disease resistance, and better adaptability to a wide range of aquatic habitats made them popular among fish farmers [7]. In Bangladesh, the production of this species in the pond was initiated in the Mymensingh district in the early 1990s [7], the most prominent fish production area. This fish is also recognized as a favorite among customers because of its low market price, particularly for the low-income residents of urban areas [7]. As a result, farming Pangasius catfish became popular among farmers quickly and was adopted in many districts in Bangladesh. Like other aquatic organisms, the physicochemical parameters of the framed ponds greatly impact the growth, survival, and production of the Pangasius catfish. As farming Pangasius catfish requires a great amount of commercial feed, fertilizers, and different chemicals, deterioration in the water quality of catfish ponds is a common fact. Most importantly, these deteriorative changes destroy the pond’s healthy and balanced composition of bacteria. Therefore, seasonal monitoring of physicochemical parameters and bacterial loads in water and sediment is critical in Pangasius catfish farms. In addition, studying the relationship between bacterial counts in fish gills and intestines with the physicochemical parameters and the pond water and sediment counts is further considered for proper management to continue optimum production.

In general, the physicochemical parameters of water, especially the temperature, pH, and transparency, show seasonal fluctuations. Seasonal changes are recognized as the most critical factor causing qualitative and quantitative changes in bacterial populations in various bodies of water [8–11]. A similar trend of bacterial fluctuations was reported in numerous studies on cultured ponds [6,12,13] and cultured fish species [12,14–16]. Nevertheless, many points remain to be considered in the case of aquaculture ponds in Bangladesh. For example, it is unknown how seasonal changes in various factors affect viable bacterial counts in pond water, sediment, and fish gills and intestines. The relationship(s) between the viable counts of pond water and sediment; and between the pond water/sediment and fish gill/intestine is not well assessed in the case of Pangasius catfish culture ponds of Bangladesh. Considering the above facts, we hypothesized that the counts of Pangasius catfish pond water and sediment would vary according to the seasons. Seasonal fluctuations in different factors will correlate with the viable counts of pond water, pond sediment, fish gills, and fish intestine. The viable counts of the pond water and sediment will be correlated with the viable counts of the gill and intestine of the Pangasius catfish. We also hypothesized that the temperature would act as the most critical driving factor among different physicochemical parameters, which will influence the viable counts of water and sediment in the culture ponds and the gills and intestines of the Pangasius catfish. Therefore, the objective of the present study is to verify the hypotheses mentioned above.

## Materials and Methods

### Study areas and duration

**Figure 1.**
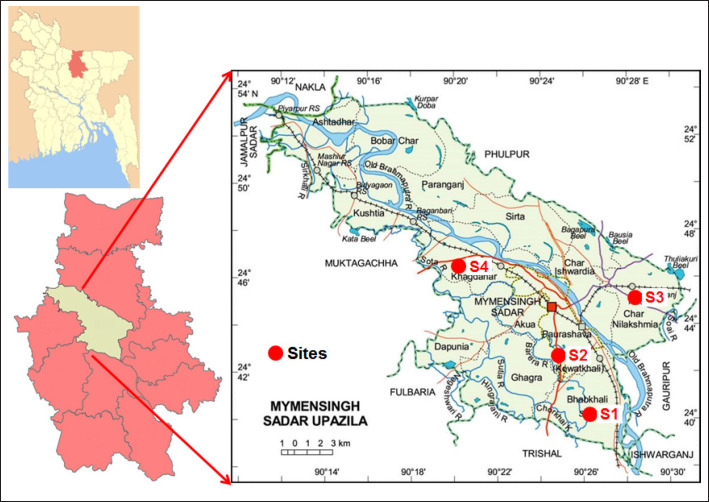
Map showing the study areas, S1 (site 1, Shutiakhali), S2 (site 2, Maskhanda), S3 (site 3, Shomvuganj), and Khagdhohor (site 4, S4) under the Sader Upazila of Mymensingh district, Bangladesh.

The study was conducted in four Pangasius catfish (*P. hypophthalmus*) producing areas such as Shutiakhali (site 1, S1), Maskhanda (site 2, S2), Shomvuganj (site 3, S3), and Khagdhohor (site 4, S4) under the Sadar Upazila of Mymensingh district (24°45’14”N and 90°24’11”E), Bangladesh (Fig. 1). Samplings were done from March 2018 to January 2019 during four different seasons, such as spring (March), summer (June), autumn (September), and winter (January).

### Sample collection

Samples were collected from twelve selected Pangasius catfish-producing ponds, three from each location. Pond water, sediment, and live Pangasius catfish were collected for microbiological assessment. Surface waters (1 foot) from three different pond locations were collected in sterile sample bottles (200 ml). Bottom sediment samples were also collected in sterile glass bottles from the three different locations of the selected ponds. The bottles with samples were kept in an icebox until carried to the laboratory for further analysis. amples Pangasius catfishes were harvested using a cast net from the sampling ponds and brought to the laboratory live. All the samplings were done in triplicate from each of the selected ponds and carried to the Fisheries Microbiology Laboratory, Department of Fisheries Technology, Bangladesh Agricultural University, Mymensingh, Bangladesh.

### Recording of physicochemical parameters

Physicochemical parameters, *viz*., ambient temperature, water temperature, water transparency, and pH of the pond waters, were measured during sampling. The ambient and surface water temperatures (°C) were monitored using an alcohol thermometer. The collected water’s pH was measured using a DF Universal pH Test Paper Strip (DF, Guangzhou, China). The transparency (cm) of the water was measured by reading the Secchi depth of the pond water using a Secchi disk.

### Determination of viable bacterial counts

#### Sample preparation

Standard plate count, expressed as colony-forming units per millimeter (CFU ml^−1^; in the case of water samples) or per gram (CFU gm^−1^; in the case of sediment, fish gill, and intestine samples), was determined. Before plating the collected samples, stock solutions were prepared and further diluted following consecutive decimal and serial dilution techniques. For water, 1 ml of the collected sample (water stock solution) was transferred with a micropipette to test tubes containing 9 ml of sterile physiological saline. For a homogeneous dilution, the tubes were thoroughly shaken using a vortex mixture to achieve a 10^−1^ dilution of the collected water sample. Following the same protocol of decimal dilution, the 10^−2^, 10^−3^, and 10^−4^ dilutions of the samples were achieved consecutively. For sediment, 1 gm of the wet sample was weighed and homogenized in 200 ml of sterile diluent (physiological saline) to prepare the first stock solution. This solution was then serially diluted following the above protocol to achieve the 10^−1^ to 10^−4^ dilutions.

Sampled live fish were sacrificed and dissected aseptically to collect their gills and intestines. These collected parts (gills and intestines) were then mixed and ground in a sterile mortar, aseptically and carefully. One gram of the ground sample was then suspended in a bottle containing 200 ml of sterile physiological saline and mixed thoroughly (by shaking and vortexing). Following the similar processes mentioned above, several dilutions of 10^−1^, 10^−2^, 10^−3^, and 10^−4^ were made.

#### Plating and incubation

The diluted samples (10^−3^ and 10^−4^) were then spread plated in duplicate in previously prepared plate count agar (HiMedia, Mumbai, India) plates. From sample solutions of different dilutions, 0.1 ml samples were taken by a micropipette and transferred aseptically onto the agar plates, which were then spread homogenously and carefully onto the surface using a sterile L-shaped glass rod until the sample was dried up. The plates with the cultures were then turned upside down and kept at 30°C for 48 h.

#### Counting and calculation

A Quebec dark field colony counter (Leica, Buffalo, New York) equipped with a guide plate ruled in square centimeters was used to count the colonies. Plates containing 30–300 colonies were considered to calculate viable bacterial counts by using the following formulae:

For water samples (CFU ml^−1^),

= No. of colonies on Petri dish × 10 × dilution factor

For sediment samples (CFU gm^−1^),


$$\frac{\mathrm{No}.\;\mathrm{of}\;\mathrm{colonies}\;\mathrm{on}\;\mathrm{petridish}\;\times \;10\;\times \;\mathrm{dilution}\;\mathrm{factor}\;\times \;\mathrm{volume}\;\mathrm{of}\;\mathrm{stock}\;\mathrm{solution}}{\mathrm{Wt}.\;\mathrm{of}\;\mathrm{the}\;\mathrm{sediment}\;\mathrm{sample}\;\mathrm{(gm)}}$$

For fish gill/ intestine samples (CFU gm^−1^),


$$\frac{\mathrm{No}.\;\mathrm{of}\;\mathrm{colonies}\;\mathrm{on}\;\mathrm{petridish}\;\times \;10\;\times \;\mathrm{dilution}\;\mathrm{factor}\;\times \;\mathrm{volume}\;\mathrm{of}\;\mathrm{stock}\;\mathrm{solution}}{\mathrm{Wt}.\;\mathrm{of}\;\mathrm{the}\;\mathrm{fish}\;\mathrm{sample}\;(\mathrm{gill}/\mathrm{intestine})\;\mathrm{(gm)}}$$

### Statistical analyses

Using Microsoft Excel 2010, the collected and generated data for physicochemical parameters and viable bacterial counts were plotted and analyzed. The box plots for physicochemical parameters were generated using the R software (version 4.1.2) with the package “ggplot2” [17]. Statistical analysis for Pearson’s correlation between the physicochemical parameters and viable bacterial counts was performed by International Business Machines Corporation Statistical Package for the Social Sciences Statistics software (version: 28.0.0.0). Redundancy analysis (RDA) was also conducted using the “Vegan” package in R software [18] based on the log_10_ values of the viable counts and physicochemical parameters. To assess the similarities or dissimilarities of the viable counts at different seasons and locations, a heatmap with clustering analysis was also performed using the R software with the “pheatmap” package [19] based on the log_10_ values of the viable counts.

## Results

### Physicochemical parameters of Pangasius catfish pond waters

**Figure 2.**
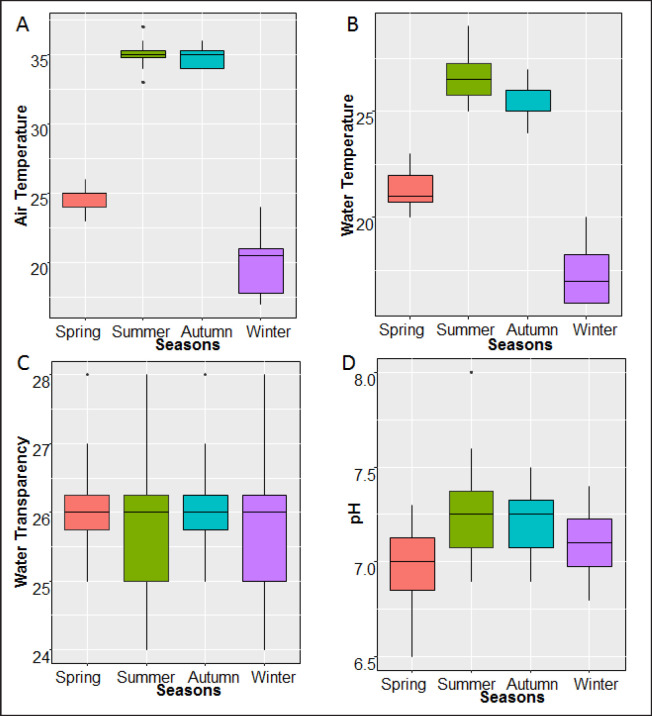
Box plots showing the seasonal fluctuations in different physicochemical parameters; A = ambient temperature, B = water temperature, C = water transparency, and D = pH of the waters of studied Pangasius catfish ponds under the Sadar Upazila of Mymensingh district, Bangladesh.

Seasonal fluctuations in different physicochemical parameters, *viz*., ambient temperature, water temperature, water transparency, and pH of the studied Pangasius catfish ponds, were recorded. The average values of these parameters are determined for different seasons and sites and presented in box plots (Fig. 2).

The ambient and water temperatures of different sampling sites and seasons fluctuated from as high as 37°C and 29°C, respectively, in summer to as low as 17°C and 16°C in winter. However, the temperatures were almost similar in different sampling sites within a particular season. During the spring season, the average ambient temperature was 25°C while the average water temperature was 21°C. During summer, the ambient temperature increased to 35°C and the water temperature to 27°C. The average ambient and water temperature were almost similar to summer during autumn, just decreasing by 1°C. However, in winter, the average ambient temperature decreased to 20°C and the water temperature to 17°C. Regardless of the sampling seasons and sites, the water transparencies were almost similar in different studied ponds, about 26 cm. Similarly, the pH values were almost stable to neutral (about seven) in different ponds of the studied sites throughout the study periods.

### Viable bacterial counts of Pangasius catfishes and ponds waters

**Figure 3.**
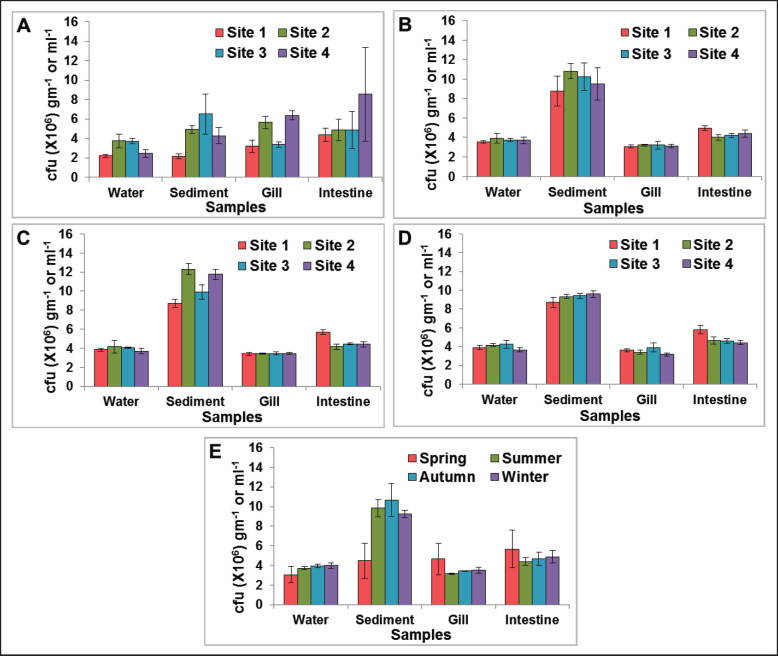
Graphs showing the seasonal fluctuations in viable counts of bacteria of pond water, sediment, fish gill, and intestine samples collected from different locations of studied Pangasius catfish ponds under the Sader Upazila of Mymensingh district, Bangladesh; A = season spring, B = season summer, C = season autumn, D = season winter, and E = average counts of different sampling locations at different seasons.

Averages of viable bacterial counts of the selected pond waters, pond sediments, fish gills, and fish intestines of the collected samples at different seasons are presented in Figure 3. In general, the counts of the gill and water were relatively lower compared to counts of the intestine and sediment (Fig. 3). Within a season, fluctuations in the counts of different samples among the studied sites were more recognizable in the case of spring compared to those of other seasons. In spring samples (Fig. 3A), site 1 had the lowest counts for all types of samples; the counts in water, sediment, gill, and intestine were, respectively, 2.2 × 10^6^ CFU ml^−1^, 2.16 × 10^6^ CFU gm^−1^, 3.2 × 10^6^ CFU gm^−1^, and 4.39 × 10^6^ CFU gm^−1^. During this season (spring), the maximum average counts of water, sediment, gill, and intestine were 3.75 × 10^6^ CFU ml^−1^ in site 2, 6.52 × 10^6^ CFU gm^−1^ in site 3, 6.37 × 10^6^ CFU gm^−1 ^in site 4, and 8.55 × 10^6^ CFU gm^−1 ^in site 4, respectively (Fig. 3A).

During the summer, within a particular sample type, no remarkable variations were obtained in the counts of different sites. The highest counts of the water, sediment, gills, and intestine were 3.9 × 10^6^ CFU ml^−1^ in site 2, 10.8 × 10^6^ CFU gm^−1 ^in site 2, 3.2 × 10^6^ CFU gm^−1 ^in both sites 2 and 3, and 4.95 × 10^6^ CFU gm^−1^ in site 1. On the other hand, the lowest counts were 3.55 × 10^6^ CFU ml^−1^ in site 1, 8.75 × 10^6^ CFU gm^−1 ^in site 1, 3.05 × 10^6^ CFU gm^−1 ^in site 1, and 4.0 × 10^6^ CFU gm^−1^ in site 2 for water, sediment, gill, and intestine samples, respectively (Fig. 3B).

Similar tendencies were also found during the autumn and winter. No remarkable differences were gathered in the counts of different sites within a particular sample type. In autumn, the water counts varied from 3.7 × 10^6^ CFU ml^−1^ in site 4 to 4.15 × 10^6^ CFU ml^−1^ in site 2, and the counts of sediment varied from 8.7 × 10^6^ CFU gm^−1^ in site 1 to 12.3 × 10^6^ CFU gm^−1 ^in site 2. During the same season, the counts of gill varied from 3.4 × 10^6^ CFU gm^−1 ^in both sites 1 and 2 to 3.45 × 10^6^ CFU gm^−1 ^in both sites 3 and 4, and the counts of the intestine varied from 4.15 × 10^6^ CFU gm^−1 ^in site 2 to 5.7 × 10^6^ CFU gm^−1 ^in site 1 (Fig. 3C). While in winter, the counts varied from 3.65 × 10^6^ CFU ml^−1^ in site 4 to 4.25 × 10^6^ CFU ml^−1^ in site 3 in the water and 8.7 × 10^6^ CFU gm^−1 ^in site 1 to 9.6 × 10^6^ CFU gm^−1 ^in site 4 in the sediment. In gill samples, the counts ranged from 3.15 × 10^6^ CFU gm^−1^ in site 4 to 3.9 × 10^6^ CFU gm^−1^ in site 3, and in intestine samples, the counts ranged from 4.4 × 10^6^ CFU gm^−1^ in site 4 to 5.8 × 10^6^ CFU gm^−1^ in site 1 (Fig. 3D).

The average counts of all the sites for a particular sample in a particular season were also calculated. Except for spring, the counts of a specific sample were consistent across seasons. The spring counts were lower than in other seasons in the cases of water and sediment but higher in the gill and intestine (Fig. 3E).

### Relationships among the viable bacterial counts and physicochemical parameters

Pearson’s correlation was performed to establish the relationship between the viable counts of bacteria and the physicochemical parameters of the studied ponds (Table 1). No significant correlations were established between the physicochemical parameters and viable bacterial counts. Water temperature showed a weak to moderately positive correlation with viable counts of pond water and sediment (*r* = 0.036 and *r* = 0.309, respectively) and a weak negative correlation with viable counts of gill and intestine (*r* = −0.284 and *r* = −0.253, respectively). However, significant positive correlations were found among the viable counts of water and sediment (*r* = 0.78) and the gill and intestine (*r* = 0.738). A significant negative correlation was observed between viable counts of pond sediment and fish gill (*r* = −0.520), while moderate negative correlations were observed between the viable counts of the pond (water and sediment) and fish (gill and intestine) (Table 1).

**Table 1.** Pearson’s correlation between the viable counts of bacteria and physicochemical parameters, and between the viable counts of pond (water and sediment) and fish (gill and intestine) of the studied Pangasius catfish ponds.

	Ambient temperature	Water temperature	Water transparency	pH	Viable counts of water	Viable counts of sediment	Viable counts of gill	Viable counts of the intestine
Ambient temperature Water temperature Water transparency pH Viable counts of water Viable counts of sediment Viable counts of gill Viable counts of the intestine	1	0.971**	0.117	0.399	0.119	0.445	−0.302	−0.265
		1	−0.02	0.287	0.036	0.309	−0.284	−0.253
			1	0.169	−0.106	0.007	0.23	0.044
				1	0.274	0.45	−0.143	0.005
					1	0.780**	−0.353	−0.483
						1	−0.520*	−0.478
							1	0.738**
								1

** Correlation is significant at the 0.01 level (2-tailed).

* Correlation is significant at the 0.05 level (2-tailed). Number of variables (*N*) = 16.

### Responses of viable bacterial counts to the physicochemical parameters

**Figure 4.**
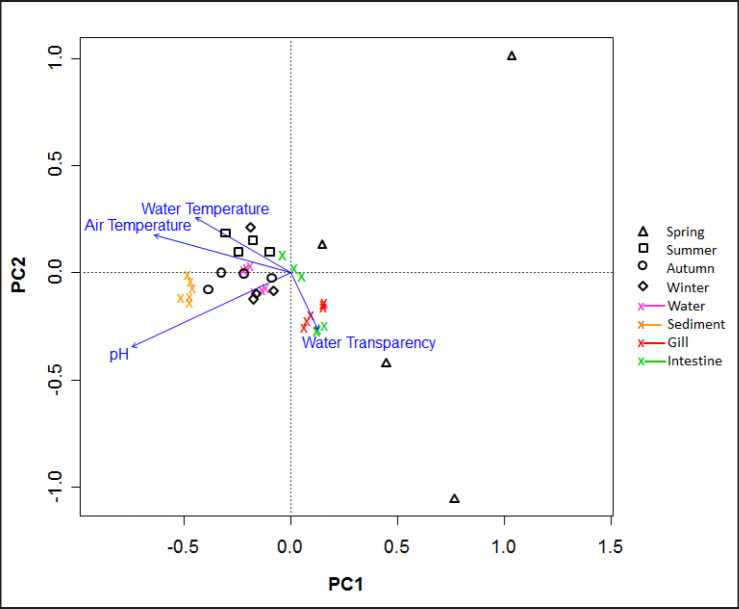
RDA plot showing the responses of viable counts of bacteria to the physicochemical parameters of the water of the studied Pangasius catfish ponds.

RDA was carried out to assess the responses of viable counts of bacteria to the physicochemical parameters of the studied Pangasius catfish ponds. The driving factor(s) that controlled the viable counts of bacteria in the ponds was also evaluated (Fig. 4). Except for the spring season, the RDA plot shows that the viable counts of four locations were closely clustered, indicating similarities at a location scale. The viable counts of the sediment samples were closely clustered. More or less similar tendencies were observed in the cases of water and gill samples, indicating relatively less fluctuation in viable counts within a particular sample type. The intestine samples were separately arranged, indicating more seasonal fluctuations in viable counts in the case of intestine samples (Fig. 4).

The RDA plot also indicated that ambient temperature, water temperature, and pH were closely related to the viable bacterial counts of all the samples, indicating their influences. Water transparencies were close to the viable counts of gill samples and most of the intestine samples (Fig. 4). To verify the degree of influence by different factors and to determine the driving factor(s) to change the viable counts of bacteria, a permutation test was done. The results showed that none of the physicochemical parameters was a significant contributor. Among the parameters, the pH was primarily influenced by clustering (*r*2 = 0.23 and *p* = 0.17, based on 1,000 permutations). Ambient temperature (*r*2 = 0.15 and *p* = 0.40, based on 1,000 permutations) and water temperature (*r*2 = 0.09 and *p* = 0.63, based on 1,000 permutations) had relatively fewer influences than water pH. Among the studied parameters, water transparency (*r*2 = 0.03 and *p* = 0.88, based on 1,000 permutations) had the least impact on the viable counts of bacteria (Table 2). However, the seasons as a whole showed a significant effect on the viable counts (*r*2 = 0.42 and *p* = 0.001, based on 1,000 permutations) above the 99.9% confidence level, i.e., viable counts fluctuated seasonally. The sampling locations had no significant effects (*r*2 = 0.13 and *p* = 0.89, based on 1,000 permutations), indicating viable counts were similar in different locations within a season (Table 2).

**Figure 5.**
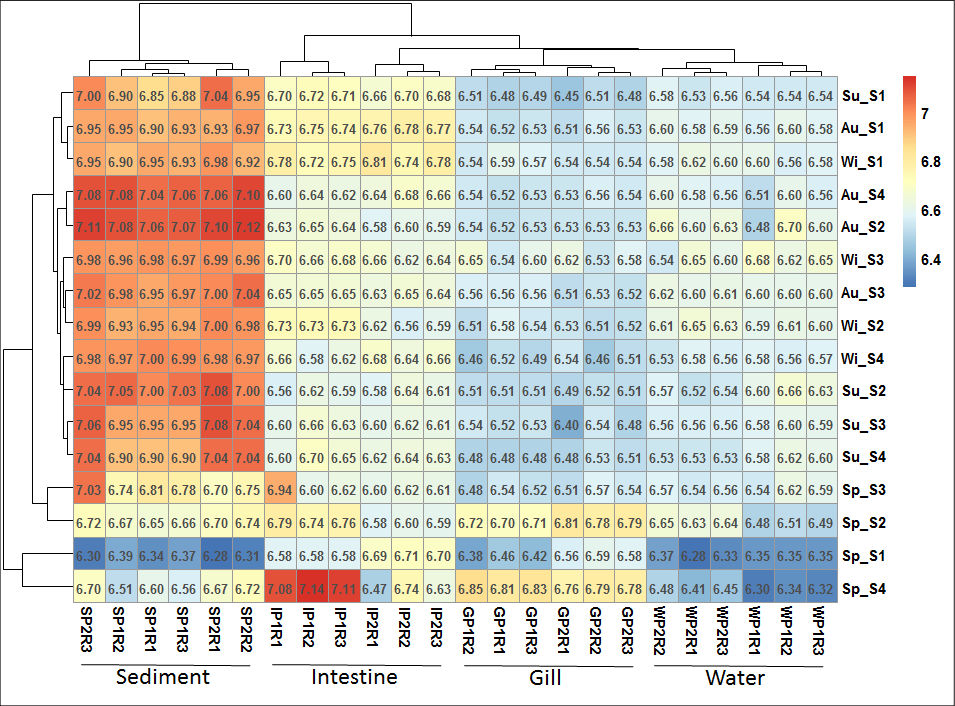
Heatmap coupled with a cluster analysis showing variations of bacterial counts of different samples at seasons and sampling locations of the studied Pangasius catfish ponds. In the figure vertically, Sp = spring, Su = summer, Au = autumn, Wi = winter; S1, S2, S3, and S4 = site 1, site 2, site 3, and site 4, respectively; horizontally, W, S, G, and I = water, sediment, gill, and intestine, respectively; P1 and P2 = pond 1, pond 2; and R1, R2, and R3 = replication 1, replication 2, and replication 3, respectively.

To evaluate the similarities and dissimilarities of the bacterial counts of different samples at different seasons and sampling sites, heatmap generation coupled with clustering analysis was conducted (Fig. 5). While considering the clustering of the samples on a seasonal scale, most of the samples were found to align closely according to the seasons. However, the samples of summer, autumn, and winter of site 1 were aligned together at a separate clade indicating similarities in viable counts of different samples of that location during most of the seasons (Fig. 5). On the other hand, counts of different samples (water, sediment, gills, and intestine) were aligned clearly and separately according to the sample types. Sediment samples were aligned at a different clade near the intestine samples. Samples of water and gill were aligned at a single clade, meaning similarities in their counts (Fig. 5).

**Table 2.** Permutation test for RDA based on the log_10_ values of the viable counts and physicochemical parameters of the waters of the studied Pangasius catfish ponds at seasonal scales.

Parameters	PC1	PC2	r2	Pr (>r)
Ambient temperature	−0.96416	0.26530	0.1524	0.4036
Water temperature	−0.86389	0.50368	0.0919	0.6314
Water transparency	0.43559	−0.90015	0.0303	0.8791
pH	−0.90600	−0.42327	0.2330	0.1738
Goodness of fit				
Seasons	-	-	0.4150	0.000999 ***
Sampling sites	-	-	0.1334	0.886114

Permutation: free, Number of permutations: 1,000.

Signifiance codes: 0 “***” 0.001 “**” 0.01 “*” 0.05 “.” 0.1 “ “ 1.

## Discussion

Responses of viable counts of the Pangasius catfish (*P. hypophthalmus*) (gill and intestine) to some physicochemical parameters and viable bacterial counts of cultured ponds (water and sediment) were assessed on a seasonal scale. We hypothesized that the viable counts of the pond (water and sediment) and fish (gill and intestine) would vary according to the seasons and correlate with the physicochemical parameters. We also hypothesized that among different physicochemical parameters, the temperature would act as the most important driving factor to influence viable counts of water and sediment in the ponds and the gills and intestines of the Pangasius catfish.

The values of the physicochemical parameters of this study are similar to Abedin et al. [20]. However, among the evaluated physicochemical parameters, temperatures fluctuated mostly between different seasons but were almost similar among different sampling sites within a particular season. The water transparency and pH were similar in different studied ponds regardless of the seasons and locations (Fig. 2); similar findings were also reported. However, averages of viable bacterial counts in the studied pond waters, pond sediments, fish gills, and fish intestines varied seasonally (Fig. 3). Seasonal changes influenced the physicochemical parameters of different water bodies, resulting in qualitative and quantitative changes in bacterial populations [8–11]. In the case of fishponds, a similar trend in the fluctuation of bacterial abundance was reported [6,12,13,21], which ultimately influences the bacterial flora of the cultured fish species [12, 14–16]. The bacterial load of the pond water was reported to vary seasonally [6]. Increased bacteria load was reported to be related to increased water temperature [22–24]. Our study established a positive correlation between temperature and the viable counts of pond water and sediment (Table 1), indicating a higher bacterial load in pond water and sediment during the warmer seasons. This may be because of the higher rate of growth by the many mesophilic bacteria near the optimum growth temperature [25] as measured in studied ponds, especially during the summer and spring (Fig. 2). Bacterial loads of the pond sediment were also reported fluctuating based on the seasonal changes; low temperature resulted in lower bacterial loads [13]. Thus, lower bacterial loads in the sediment samples of our studied ponds may be because of the reduced growth rate of bacteria during the winter season (Fig. 2). However, regardless of the seasons, the viable bacterial counts were higher in the sediment samples compared to the waters (Fig. 3). This is maybe because of the higher organic enrichment in the sediments of the pond water, which is due to the accumulation of dead organic matter, uneaten feed, fish excretory materials in the bottom sediments. The increased bacterial population was reported in benthic environments with higher loads of organic matter [26,27]. Many studies reported higher bacterial loads in fish during periods of increased water temperature [15,16,23,24]. However, the scenario was different in this study. In the Pangasius catfish ponds, we observed a weak negative correlation between water temperature and viable counts of gill and intestine of Pangasius catfish (Table 1). In the winter season in Bangladesh, the volume of fishpond water reduces, and almost no rainfall occurs, which may increase the colonization of bacteria in fish.

In general, the abundance and composition of bacterial flora in fishponds depend not only on the types and geographical location but also on the water’s physicochemical parameters (especially the content of organic and inorganic materials, pH, temperature, turbidity, etc.). Competition among the organisms for nutritional purposes is also an essential factor responsible for quantitative and qualitative fluctuations in bacterial flora. The temperature was reported as the most influential diver that affects the microbiomes in aquatic environments [28,29] and fish and other aquatic organisms [30,31]. We identified none of the assessed physicochemical parameters as significant contributors to viable bacteria counts in the Pangasius catfish pond. However, among the parameters, the permutations test indicated that the influences of the pH were relatively higher than the water temperature and water transparencies. However, a significant seasonal impact was observed on the viable bacterial counts of the Pangasius catfish ponds (Table 2); as reported, the bacterial community composition is primarily modified due to the seasonal variability in the intensive freshwater aquaculture [12].

## Conclusion

The viable bacterial counts of the Pangasius catfish ponds varied significantly among the seasons. The counts of the ponds (water and sediment) showed a weakly positive correlation with the temperature and a weakly negative correlation with the fish counts (intestine and gill). None of the assessed physicochemical parameters found significant drivers to influence the viable bacterial counts of the Pangasius catfish ponds.

## List of Abbreviation

RDA, Redundancy analysis.
